# Evolution of deep learning for structural magnetic resonance imaging analysis in Alzheimer’s disease: from slice-level classification to brain structure modeling

**DOI:** 10.3389/fnhum.2026.1958462

**Published:** 2026-09-30

**Authors:** Yunchen Chen, Tingshan Liu, Guanxun Cheng

**Affiliations:** 1 Shenzhen University Medical School, Shenzhen University, Shenzhen, China; 2 Department of Medical Imaging, Peking University Shenzhen Hospital, Shenzhen, China; 3 Key Laboratory of Biomedical Imaging Science and System, State Key Laboratory of Biomedical Imaging Science and System, Chinese Academy of Sciences, Shenzhen, China

**Keywords:** Alzheimer’s disease, clinical translation, convolutional neural network, deep learning, mild cognitive impairment, structural magnetic resonance imaging, transformer

## Abstract

Structural magnetic resonance imaging (sMRI) depicts Alzheimer’s disease (AD)-related atrophy noninvasively and has become a common input for deep-learning studies of diagnosis and progression. For this structured narrative Mini Review, we searched PubMed/MEDLINE, the Web of Science Core Collection, and IEEE Xplore through 31 August 2026. We then purposively selected recent studies that were informative about model design or evaluation. The review covers two-dimensional convolutional neural networks, three-dimensional and hybrid volumetric architectures, transformers, and foundation models, together with longitudinal, multimodal, and graph-based extensions. Across these approaches, validity depends on clinically meaningful target labels, preprocessing that excludes test-set information, participant-level data separation, and evaluation beyond discrimination. Two-dimensional methods remain useful when data or computing resources are limited, although they do not preserve continuous whole-volume context. Three-dimensional and hybrid models incorporate more volumetric information, but anatomical fidelity still depends on representation and validation. Current AD-specific studies do not show that transformers or foundation models consistently outperform convolutional or hybrid alternatives. Stronger clinical evidence will require external testing, calibrated and uncertainty-aware predictions, transparent reporting, and prospective assessment within the intended workflow.

## 1 Introduction

Structural magnetic resonance imaging (sMRI) offers a noninvasive view of Alzheimer’s disease (AD)-related atrophy and is widely used in imaging assessment (Chouliaras and O’Brien, 2023). Earlier reviews summarized deep learning across neuroimaging and other biomarkers, including subject-, region-of-interest (ROI)-, patch-, and slice-level representations (Goenka and Tiwari, 2021; Frizzell et al., 2022; Khojaste-Sarakhsi et al., 2022; Xu et al., 2023). Here, we focus on how the chosen representation and spatial model affect leakage control, validation, and the credibility of clinical claims. We use “brain structure modeling” to mean either preservation of volumetric anatomical context or learning of spatial relations among slices, patches, voxels, regions, or participants. The term does not imply that every 3D or graph model is anatomically explicit or causal. We examine 2D, 3D/hybrid, and global-dependency or pretraining approaches, as well as longitudinal, multimodal, and graph-based extensions. These are overlapping design choices rather than successive stages in a single technical trajectory.

## 2 Methods

This structured narrative Mini Review traces technical developments and was not conducted as an exhaustive systematic review or meta-analysis. We searched PubMed/MEDLINE and IEEE Xplore on 1 September 2026 and the Web of Science Core Collection on 2 September 2026. Coverage was limited through 31 August 2026; records were retained when the publication date or, where applicable, the Early Access date was on or before that cutoff. The searches yielded 1,361 records: 356 from PubMed/MEDLINE, 372 from IEEE Xplore, and 633 from the Web of Science Core Collection. Comparison of DOI, normalized title, authors, year, and publication version consolidated 414 duplicate-source records, leaving 947 unique candidate citations. Early Access and version-of-record entries were counted once; conference and journal reports were merged only when they described the same study. These counts describe the mapping pool rather than a PRISMA study flow. Supplementary Table S1 provides the executed strategies and logs.

Most representative studies were published between 1 January 2021 and 31 August 2026. Earlier sources were retained when they established a method, dataset, or evaluation principle needed for the review. The purposive evidence set addresses representation, spatial and longitudinal modeling, training, and clinically relevant evaluation. We prioritized English-language, peer-reviewed full reports that used sMRI directly in AD or MCI tasks. Multimodal studies were included only when sMRI was an explicit input. Review articles were used for context and to identify additional primary studies. Citation counts, journal metrics, and headline accuracy were not used as selection criteria. The rationale for each highlighted study is reported in Supplementary Table S2.

We synthesized the evidence qualitatively because the studies differed substantially in task, target label, preprocessing, data unit, split, and validation design. Pooling their performance or ranking architectures would therefore be difficult to justify. Table 1 instead compares one contextual review and nine illustrative primary reports by cohort size, task, validation, contribution, and claim boundary. Participant counts are distinguished from repeated scans and derived slices. OpenAI Codex in ChatGPT assisted with multi-field de-duplication, preliminary relevance triage, terminology checks, and language editing; the authors made all final decisions on study selection, data extraction, interpretation, and wording.

**Table 1:** Representative and foundational studies (2018–2026) of deep learning for structural MRI analysis in Alzheimer’s disease, with study-specific strengths and limitations.

Study and role	Cohort, task, and validation	Method and principal contribution	Main evidence boundary
Parisot et al. (2018) Foundational population GCN	ADNI subset: 540 participants with early/late MCI and 1,675 longitudinal acquisitions; 289 participants were classified as converters. MCI-conversion prediction used stratified 10-fold cross-validation, with all visits from one participant retained in one fold.	A population graph combines volumes of 138 segmented structures as imaging features with phenotypic and within-participant relations as edges, enabling semi-supervised graph convolution.	Single public cohort and hand-engineered regional volumes; graph construction encodes assumptions and the semi-supervised/transductive setting may expose test-node context. It is not an end-to-end voxel-level anatomical GNN or prospective clinical validation.
Goenka and Tiwari (2021) Review/cross-modal context	Narrative review of neuroimaging, cerebrospinal-fluid, blood/urine, and genetic biomarkers; no primary cohort.	Contrasts subject-, ROI-, and patch-level 3D representations and links biomarker choice to model design.	Broader than sMRI; heterogeneous modalities and tasks preclude direct performance ranking.
Kang et al. (2021) 2D CNN	ADNI-1 baseline T1 sMRI from 798 participants (187 AD, 382 MCI, and 229 CN); binary AD/CN, AD/MCI, and MCI/CN tasks; internal evaluation.	Selected coronal gray-matter-density slices with VGG16, ResNet50, and a GAN-discriminator ensemble; combines multi-slice and multi-model aggregation.	Preselected 2D slices do not preserve whole-volume continuity; results depend on segmentation, slice selection, and internal ADNI validation.
Zhu et al. (2021) 3D multi-instance attention	1,689 baseline sMRI participants: 1,193 from ADNI (389 AD, 172 pMCI, 232 sMCI, and 400 NC) and 496 from AIBL (79 AD, 17 pMCI, 93 sMCI, and 307 NC); four binary tasks (AD/NC, pMCI/sMCI, pMCI/NC, and sMCI/NC). ADNI was split 80:20 for development and held-out testing, with five-fold cross-validation within the development portion; the trained model was also evaluated on independent AIBL data.	Dual-attention multi-instance learning combines local instances with participant-level evidence and highlights discriminative regions.	Retrospective public cohorts; independent AIBL testing supports cross-dataset robustness but does not establish clinical calibration or prospective utility; attention maps remain associative.
Lian et al. (2022) Hybrid 3D network	ADNI-1 (n = 821), ADNI-2 (n = 637), and AIBL (n = 519) sMRI; AD/NC diagnosis and pMCI/sMCI conversion prediction; development on ADNI-1 with testing on ADNI-2 and AIBL.	Weakly supervised disease-region localization with a multibranch hybrid network links regional localization to multilevel feature fusion.	Retrospective public cohorts; testing on ADNI-2 and AIBL strengthens transportability evidence, but calibration and prospective clinical utility were not established; weakly supervised localization remains associative and requires biological corroboration.
Goenka and Tiwari (2023) Multimodal 3D comparison	ADNI T1-weighted sMRI and AV-45 PET; binary and three-class diagnosis; internal evaluation. The accessible primary report did not provide a clearly verifiable independent participant denominator. The 4,315-image/843-participant figures in its related-work discussion refer to Gupta et al. (2013), not to the Goenka–Tiwari experimental cohort.	Compares 3D subject-, patch-, and reconstructed slice-based inputs within one multimodal pipeline and provides code.	Representation and modality effects are not isolated, so sMRI-only or cross-cohort superiority cannot be inferred. Cohort size and participant-disjoint validation cannot be asserted from the accessible primary report.
Chattopadhyay et al. (2024) Architecture/biomarker comparison	Overall ADNI pool: 1,847 participants with amyloid-PET labels. CNN experiments: 762 participants. Transformer training: 1,290 scans from 845 participants; validation: 276 scans; test: 275 scans, excluding participants represented in training/validation. Task: amyloid positivity.	Compares 2D CNN, 3D CNN, hybrid CNN, and two neuroimage-transformer variants within one biomarker-defined task.	CNN and transformer samples differ, preventing a strictly matched architecture-only comparison. Retrospective ADNI evidence; amyloid positivity is not clinical AD diagnosis, and subgroup performance varied.
Dhinagar et al. (2025) PEFT / masked autoencoder	T1-weighted MRI; multiple neuroimaging benchmarks, including AD classification with 258 training scans; task-specific internal evaluation.	Compares parameter-efficient fine-tuning with full fine-tuning and training from scratch, greatly reducing the trainable parameter fraction while retaining competitive task performance.	Conference-proceedings evidence; small AD training set and heterogeneous tasks; external clinical validation, calibration, and workflow value were not established.
Lin et al. (2025) Vision–language foundation model	ADNI: 841 participants; reported training/validation/test partitions of 2,403/332/663 scans, respectively. External zero-shot evaluation: 652 HABS-HD participants. Tasks include AD/MCI/CN classification and MMSE prediction. See the count-consistency note below.	ADLIP aligns a 3D DenseNet encoder with Bio_ClinicalBERT using clinical-text supervision and contrastive learning. Zero-shot inference compares MRI with candidate label prompts; clinical text is training supervision, not necessarily patient-specific inference input.	External and subgroup tests support transfer. Internal participant-disjoint splitting is not specified; longitudinal analysis selected correctly classified scans. Retrospective results do not establish prospective workflow benefit or comprehensive fairness.
Bricaud and Masala (2026) Architecture benchmark	639 ADNI participants contributed 3,134 longitudinal scans. CN/MCI/AD classification with scan-based internal splitting; test evaluation used 627 scans and 6,353 derived slices.	Compares 15 CNN, transformer, and hybrid architectures and separates slice- from scan-level predictions within one preprocessing and aggregation pipeline.	The authors explicitly state that participant-level separation was not enforced: longitudinal visits from one person could occur across partitions. The benchmark therefore does not establish generalization to unseen participants or clinical centers.

The studies were chosen to illustrate the main technical questions discussed in this narrative Mini Review. Recent evidence was prioritized, while Parisot et al. (2018) was retained as a foundational example of graph-based population modeling. The set is neither exhaustive nor derived from a PRISMA process. Direct cross-study performance ranking would be inappropriate because tasks, labels, preprocessing, data units, splits, and validation designs differ. AD, Alzheimer’s disease; ADNI, Alzheimer’s Disease Neuroimaging Initiative; AIBL, Australian Imaging, Biomarkers and Lifestyle study; CN, cognitively normal; CNN, convolutional neural network; GAN, generative adversarial network; GCN, graph convolutional network; HABS-HD, Health and Aging Brain Study–Health Disparities; MCI, mild cognitive impairment; MMSE, Mini-Mental State Examination; MRI, magnetic resonance imaging; NC, normal control (source terminology); PEFT, parameter-efficient fine-tuning; PET, positron emission tomography; pMCI, progressive MCI; ROI, region of interest; sMCI, stable MCI; sMRI, structural MRI. Count-consistency note for Lin et al. (2025): the source text reports 3,396 ADNI scans, whereas its Table 1 partitions (2,403 + 332 + 663) sum to 3,398. The partition counts are reproduced as reported; no reconciled aggregate total is asserted.

## 3 Data sources, input representations, and sources of bias

Public research cohorts provide most data used in sMRI deep-learning studies. The Alzheimer’s Disease Neuroimaging Initiative (ADNI) contains longitudinal, multicenter, and multimodal data (Veitch et al., 2022). The Open Access Series of Imaging Studies (OASIS) provides cross-sectional and longitudinal T1-weighted MRI with clinical characterization (Marcus et al., 2007, 2010). Repeated use of these cohorts facilitates comparison but concentrates evidence in research populations that share acquisition and preprocessing conventions.

### 3.1 Preprocessing dependence and reproducibility

Inputs range from manually defined ROIs to registered whole-brain volumes and minimally processed native-space images. ROI analysis reduces dimensionality but can miss distributed atrophy (Folego et al., 2020; Frizzell et al., 2022). Performance is conditional on skull stripping, bias-field correction, registration, tissue segmentation, resampling, intensity normalization, and quality control. Participant separation must precede augmentation and any fitted normalization, feature selection, atlas construction, or harmonization; visits, slices, and patches from one person must remain in one fold (Wen et al., 2020; Yagis et al., 2021). Reports should specify software, versions, parameters, template or native space, failed-scan handling, and any use of test data during harmonization (Guan et al., 2021).

### 3.2 Diagnostic targets and intended use

AD/CN, MCI/CN, progressive/stable MCI, amyloid positivity, and cognitive-score prediction are distinct targets. Clinical labels describe a syndrome and may contain substantial heterogeneity, whereas biomarker labels indicate pathology without by themselves establishing dementia. Progression studies should define the index visit, conversion horizon, censoring, and rules for reversed diagnoses. Intended use also matters. A diagnostic aid is applied to symptomatic patients; screening is offered to a broader asymptomatic or at-risk population; and early detection refers to timing and does not necessarily imply screening. Prevalence affects positive and negative predictive values, referral burden, and potential harm. Accuracy obtained from a balanced case–control dataset is therefore insufficient to support an unqualified screening claim (Chouliaras and O’Brien, 2023; Veitch et al., 2022; Chattopadhyay et al., 2024).

## 4 Evolution of spatial modeling with deep learning

Each modeling strategy balances computational burden against the amount and type of anatomical or relational context it represents. Table 1 distinguishes participants from repeated scans and derived slices because image count is not a measure of independent sample size.

### 4.1 2D CNNs: transfer-learning-driven slice-level classification

Two-dimensional convolutional neural networks (2D CNNs) can use natural-image pretraining when neuroimaging data or computing resources are limited. Kang et al. (2021) analyzed baseline T1-weighted MRI from 798 ADNI participants. They selected 11 coronal gray-matter-density slices, trained VGG16, ResNet50, and a generative adversarial network discriminator, and combined the slice-level decisions by majority voting before model ensembling. The resulting transfer-learning pipeline addressed AD/CN, AD/MCI, and MCI/CN classification. Predictions were aggregated at the participant level, and validation remained internal to ADNI.

Aggregating several slices samples information from multiple locations without preserving continuous whole-brain geometry. Adjacent-slice and 2.5D designs offer an intermediate representation (Soelistiono, 2026). Their validity depends on anatomically defensible slice selection and participant-level aggregation. All slices and longitudinal visits from one participant should remain in the same partition, as slice-level separation can substantially inflate performance (Yagis et al., 2021).

### 4.2 3D CNNs and transitional architectures

Three-dimensional CNNs operate on volumetric neighborhoods, while patch-based models process smaller local volumes to reduce memory use. Zhu et al. (2021) evaluated DA-MIDL on baseline sMRI from 1,689 ADNI and AIBL participants. Attention-weighted multi-instance pooling combined patch features into participant-level evidence for a global classifier rather than averaging independent slice predictions. HybNet coupled weak disease-region localization with multilevel fusion (Lian et al., 2022; Table 1). Attention weights indicate association and should not be interpreted as causal anatomical explanations.

Chen et al. (2022) combined attention-weighted 2D features from three anatomical views with a whole-volume 3D-CNN branch. After overlap was removed, the sample comprised 808 ADNI-1 and 643 ADNI-2 participants. Training on ADNI-1 and testing on ADNI-2 covered AD/CN and progressive/stable MCI tasks; the reverse direction covered AD/CN only. This was cross-phase ADNI testing, not routine-care validation. Pseudo-3D convolution factorizes volumetric operations (Pei et al., 2022), and memory use depends on implementation rather than dimensionality alone (Qin et al., 2022; Xu et al., 2023). Goenka and Tiwari (2023) compared subject-, patch-, and reconstructed-slice inputs in a multimodal ADNI pipeline. Its strongest MRI-plus-PET result is not sMRI-only evidence.

### 4.3 Graph-based relational modeling

Graph methods treat participants or brain regions as nodes. Parisot et al. (2018) constructed a population graph from 540 ADNI participants with MCI (1,675 longitudinal acquisitions; 289 converters). Volumes from 138 segmented structures were imaging features, while age, sex, and within-person relations informed the edges. Ten-fold cross-validation kept each participant’s visits in one fold. The model integrates relational information but is not an end-to-end anatomical GNN. Findings depend on edge construction, and transductive settings may expose test-node context. Region graphs also depend on atlas, node, and edge definitions. Graphs can complement voxel-, patch-, or slice-based representations (Ali et al., 2025).

### 4.4 Transformer models and emerging foundation models

Self-attention connects spatially separated tokens, whereas convolution provides local inductive bias (Vaswani et al., 2017; Dosovitskiy et al., 2021; Liu et al., 2021). Chattopadhyay et al. (2024) compared CNN, hybrid, and neuroimage-transformer models for amyloid positivity, not clinical AD diagnosis. The ADNI pool contained 1,847 participants. CNN experiments used 762 participants; transformer training used 1,290 scans from 845 participants, with 276 validation and 275 test scans. Test participants were excluded from training and validation, but different samples prevent a strictly matched architecture comparison.

Bricaud and Masala (2026) evaluated 15 architectures using 3,134 longitudinal ADNI scans from 639 participants. Their test set contained 627 scans and 6,353 derived slices. Within that pipeline, selected CNN and hybrid models performed better than stand-alone transformers. The split, however, was made at the scan level, allowing visits from the same participant to enter different partitions. The benchmark therefore cannot establish performance in previously unseen patients.

Pretraining represents a separate design choice. Dhinagar et al. (2025) adapted a masked autoencoder pretrained on T1-weighted MRI by updating only a small subset of parameters; the AD experiment used 258 training scans (Table 1). Lin et al. (2025) developed ADLIP with 841 ADNI participants and evaluated transfer in 652 HABS-HD participants. A 3D DenseNet image encoder was aligned with Bio_ClinicalBERT through clinical-text supervision and contrastive learning. During zero-shot inference, MRI embeddings were compared with candidate label prompts. Patient-specific text was therefore not required at inference, and the foundation-model designation should not be taken to imply a transformer image backbone.

## 5 Evolution of training strategies under data constraints

Limited labeled data and class imbalance have encouraged greater use of pretraining. Self-supervised or masked-image pretraining, followed by full or parameter-efficient fine-tuning, can learn representations from unlabeled scans (He et al., 2022; Tang et al., 2022; Chen et al., 2023; Dhinagar et al., 2023, 2025; Lin et al., 2025). Any advantage should be demonstrated against a matched supervised baseline and on independent data rather than inferred from model size.

Augmentation strategies now extend beyond geometric transformations. Generative adversarial networks have been investigated for incomplete MRI/PET data and class imbalance (Pan et al., 2022; Huynh and Deshpande, 2024), and diffusion models are increasingly used elsewhere in medical imaging (Kazerouni et al., 2023). Direct evidence for diffusion models in AD-specific sMRI remains sparse. Evaluation of synthetic images should address anatomical validity, privacy, duplication, and preservation of disease morphology in addition to downstream accuracy.

### 5.1 Longitudinal and multimodal learning

Longitudinal models should represent within-person change instead of treating visits as independent observations. Conversion, interval risk, and change in cognition or atrophy each require a defined index time, prediction horizon, censoring rule, visit-spacing strategy, and procedure for reversed diagnoses. Baseline-to-conversion classifiers (Zhu et al., 2021; Lian et al., 2022) differ from models that ingest repeated observations. Population graphs (Parisot et al., 2018), 2.5D trajectories (Soelistiono, 2026), and vision-language representations (Lin et al., 2025) illustrate distinct strategies. All visits from one participant must remain in the same partition, and participant counts should accompany scan counts. Single-visit and change-score baselines are needed to show whether temporal modeling adds information.

Different modalities describe different aspects of disease. sMRI measures anatomy; PET and fluid biomarkers provide information about pathology; cognitive tests characterize phenotype; and demographic or clinical text adds context. Qiu et al. (2022) and Lin et al. (2025) illustrate multimodal fusion and cross-modal supervision. To demonstrate incremental value, however, ablation analyses should use the same participants, labels, splits, and clinically available inputs. Missingness may itself carry information in routine care. Models should therefore distinguish tests that are unavailable, failed, or not indicated and should be evaluated under realistic modality patterns. Completion and missing-modality methods (Pan et al., 2022; Gravina et al., 2024) require comparison with complete-case and sMRI-only baselines, with no target or test-set information entering imputation.

## 6 Evaluation requirements: beyond accuracy toward clinical evidence

Accuracy and the area under the receiver operating characteristic curve describe discrimination but do not show whether probabilities are reliable or whether performance is stable across centers, populations, labels, and scanners. Evaluation should also examine calibration, participant-level and external generalizability, subgroup performance, and errors with clinical consequences (Guo et al., 2017; Ovadia et al., 2019).

Calibration and uncertainty quantification answer related but different questions. Calibration measures the agreement between predicted probabilities and observed frequencies in a population or subgroup. Uncertainty quantification characterizes uncertainty around an individual prediction. Aleatoric uncertainty reflects irreducible ambiguity in the data, whereas epistemic uncertainty arises from limited knowledge and may increase under distribution shift (Kendall and Gal, 2017). Candidate methods include deep ensembles, Monte Carlo dropout, predictive entropy or variance, and explicit abstention. Calibration curves, Brier score, or negative log-likelihood should be reported alongside coverage-error or selective-risk analyses and out-of-distribution testing. Uncertainty should be reassessed on external data, and a single threshold should not be assumed to generalize across settings (Guo et al., 2017; Ovadia et al., 2019).

Interpretability requires more than a visualization. Grad-CAM can localize regions associated with a prediction, but it does not demonstrate causal reliance or biological validity, and the stability of its maps should be tested (Selvaraju et al., 2017; Adebayo et al., 2018). Explanations are more credible when they reproduce across cohorts, agree with independent atlases or quantitative regional measures, and are examined together with uncertainty and error patterns (Qiu et al., 2022; Wang et al., 2023; Bloch and Friedrich, 2024).

## 7 Discussion

We consider the implications of the evidence at three levels: technical modeling, validation, and clinical translation.

### 7.1 Technical level: architectural evolution and methodological trade-offs

The available evidence does not support a simple progression from 2D models to 3D models and then to transformers. A 2D approach can be appropriate when data or computing resources are limited, provided that slice selection, participant-level aggregation, and leakage control are clearly specified. A 3D model preserves volumetric neighborhoods, although anatomical fidelity still depends on preprocessing, coverage, and validation. Longitudinal, multimodal, and graph models add temporal, cross-modal, or relational information while introducing assumptions about visits, missingness, or edge definition. Reported accuracy alone cannot separate architecture effects from differences in labels, samples, and validation. Chattopadhyay et al. (2024) used different samples for different model families, whereas Bricaud and Masala (2026) allowed visits from one participant to cross partitions. Neither study supports an unrestricted ranking of architectures.

### 7.2 Evidence level: generalizability, validation, and reporting

Scanner vendor, acquisition protocol, site, preprocessing, demographic composition, comorbidity, and target definition can affect generalizability. Harmonization and domain adaptation may reduce site-related variation, but they must be fitted without test-set information and evaluated on unseen sites (Guan et al., 2021; Lei et al., 2024). A 2025 scoping review of 44 AD deep-learning studies found that 15.9% reported external validation, 79.5% did not control for confounders, and one within-study comparison lost 28 percentage points of accuracy after appropriate validation (Young et al., 2025). These review-level figures are not pooled estimates from Table 1. Broader medical-imaging studies have also shown uneven performance across demographic groups (Zong et al., 2023; Yang et al., 2024).

Future studies should define participant-level splits in advance, address major confounders, distinguish clinical from biomarker labels, and give greater weight to external and subgroup evaluation. CLAIM 2024 and FUTURE-AI provide guidance on reporting data splits, preprocessing, testing, reproducibility, robustness, fairness, usability, and explainability (Tejani et al., 2024; Lekadir et al., 2025). The scope of each clinical claim should reflect the strength of its validation rather than internal accuracy or computational efficiency alone.

### 7.3 Clinical level: clinical validation and workflow integration

Clinical deployment requires evidence across the care pathway. Qiu et al. (2022) compared retrospective MRI-only and multimodal classifiers with clinicians using simulated cases. The study shows diagnostic feasibility, but not improved patient outcomes from AI assistance. Sub-minute inference on one GPU also does not represent end-to-end turnaround. Data transfer, preprocessing, quality-control failures, hardware, and clinician review should be measured in the intended setting (Tejani et al., 2024; Lekadir et al., 2025).

Studies should state the model’s intended role, target population, and decision threshold. Before live use, a prospective silent-mode study, in which predictions are withheld from clinical decisions, can evaluate local compatibility, calibration, uncertainty, and failure handling. A predefined review pathway is needed for unusable scans and uncertain outputs. These proposals are based on general guidance for healthcare AI because comparable AD-specific trials have not yet been completed (Lekadir et al., 2025).

Subsequent live evaluations should compare clinicians working with and without AI. Relevant outcomes include changes in decisions, review time, additional investigations, and clinically consequential errors. DECIDE-AI provides guidance for reporting early clinical evaluation, safety, and human factors (Vasey et al., 2022). After deployment, monitoring should cover shifts in scanners or patient populations, subgroup performance, failure events, and model versions. Retrospective accuracy and a reduced number of trainable parameters are insufficient evidence of workflow benefit.

### 7.4 Strengths and limitations of this review

This purposive Mini Review considers spatial representation alongside longitudinal, multimodal, graph-based, and training strategies, while delimiting transformer, foundation-model, and clinical claims. The three-database search covered English-language literature and emphasized 2021–2026; studies indexed only in Scopus, Embase, or the ACM Digital Library may be underrepresented. We did not perform independent duplicate screening, formal risk-of-bias assessment, meta-analysis, or a PRISMA study flow. Table 1 cannot estimate the prevalence or overall quality of methods in the field. Repeated reliance on ADNI limits cohort independence and demographic breadth, and prospective AD-specific evidence for newer architectures remains scarce.

## 8 Conclusion

Deep learning with sMRI in AD now includes 2D, 2.5D, 3D, hybrid, graph-based, transformer, and pretrained models. Their value is determined less by architectural novelty than by valid target definitions, preprocessing that excludes test information, participant-level evaluation, and claims that match the available evidence. Longitudinal and multimodal models should demonstrate added value over simpler baselines. Foundation models are promising, but their AD-specific validation remains limited. Progress toward clinical use will depend on external testing, calibration and uncertainty assessment, transparent reporting, and prospective evaluation within real workflows.
