# Supplementary material for: Evolution of deep learning for structural magnetic resonance imaging analysis in Alzheimer’s disease: from slice-level classification to brain structure modeling
Source: Front Hum Neurosci. 2026 Sep 30;20:1958462. doi: 10.3389/fnhum.2026.1958462 (PMC13662426; doi:10.3389/fnhum.2026.1958462)
Supplement: Supplementary file 1 [file Table_1.DOCX]

**Supplementary Search Transparency and Representative Evidence Rationale**

*Evolution of Deep Learning for Structural Magnetic Resonance Imaging Analysis in Alzheimer's Disease: From Slice-Level Classification to Brain Structure Modeling*

**Scope statement.** This supplement documents the search and the rationale for the illustrative evidence set used in a structured narrative Mini Review. It improves reproducibility but does not convert the article into a systematic review. The 947 unique citations form a candidate mapping pool; they are not a PRISMA-style included-study set, and no claim of exhaustive coverage is made.

**Search and de-duplication audit**

The three database searches produced 1,361 source records: PubMed/MEDLINE, 356; Web of Science Core Collection, 633; and IEEE Xplore, 372. Multi-field consolidation identified 414 duplicate source records, leaving 947 unique candidate citations.

• Exact DOI agreement was used when available, but DOI alone was not sufficient.

• Normalized title, author names, publication year, source, and record type were checked jointly.

• Early Access and version-of-record entries were counted once. A later issue date did not create a second study when the Early Access record fell within the database cutoff.

• Conference and journal records were merged only when they reported the same study; substantively extended reports were retained and linked as related records.

• The audit recorded 447 duplicate-pair decisions, 44 nonduplicate decisions, and 29 related-report pairs retained separately. Pair-level decisions exceed the number of removed records because one duplicate group can contain more than one pair.

**Supplementary Table S1. Database-specific search strategies and search log**

| **Database** | **Executed / coverage cutoff** | **Records** | **Audit note** |
| --- | --- | --- | --- |
| PubMed/MEDLINE | 1 September 2026 1900-01-01 to 2026-08-31 (publication date) | 356 | All records exported in NBIB format; 356 PMID-tagged records verified. |
| Web of Science Core Collection | 2 September 2026 1900-01-01 to 2026-08-31 (DOP) | 633 | Core Collection indexes available in the institutional session: A&HCI, CCR-EXPANDED, ESCI, IC, CPCI-SSH, CPCI-S, SCI-EXPANDED, and SSCI. Records were exported in two plain-text batches (1–500 and 501–633). In the exported data, 218 Web of Science records carried Early Access metadata; 14 later received issue dates after the cutoff and were retained only because their Early Access dates were on or before 31 August 2026. |
| IEEE Xplore | 1 September 2026 Through 2026-08-31 under IEEE Xplore date/indexing semantics | 372 | Result-list audit comprised 308 conference papers, 59 journal articles, and 5 Early Access records. All 372 unique IEEE document identifiers were captured. |

Supplementary Table S1 (continued). Exact executed search strings

| **Database** | **Exact executed query** |
| --- | --- |
| PubMed/MEDLINE | (("Alzheimer Disease"[Mesh] OR Alzheimer*[Title/Abstract] OR "mild cognitive impairment"[Title/Abstract] OR MCI[Title/Abstract]) AND ("structural magnetic resonance imaging"[Title/Abstract] OR "structural MRI"[Title/Abstract] OR "structural MR imaging"[Title/Abstract] OR sMRI[Title/Abstract] OR "T1-weighted MRI"[Title/Abstract] OR "T1 weighted MRI"[Title/Abstract] OR "T1-weighted magnetic resonance imaging"[Title/Abstract]) AND ("Deep Learning"[Mesh] OR "deep learning"[Title/Abstract] OR "convolutional neural network"[Title/Abstract] OR "convolutional neural networks"[Title/Abstract] OR CNN[Title/Abstract] OR CNNs[Title/Abstract] OR transformer*[Title/Abstract] OR "self-supervised learning"[Title/Abstract] OR "foundation model"[Title/Abstract] OR "foundation models"[Title/Abstract])) AND ("1900/01/01"[Date - Publication] : "2026/08/31"[Date - Publication]) |
| Web of Science Core Collection | TS=(Alzheimer* OR "mild cognitive impairment" OR MCI) AND ALL=("structural magnetic resonance imaging" OR "structural MRI" OR "structural MR imaging" OR sMRI OR "T1-weighted MRI" OR "T1 weighted MRI") AND ALL=("deep learning" OR "convolutional neural network*" OR CNN OR CNNs OR transformer* OR "self-supervised learning" OR "foundation model*") AND DOP=1900-01-01/2026-08-31 |
| IEEE Xplore | (Alzheimer* OR "mild cognitive impairment" OR MCI) AND ("structural magnetic resonance imaging" OR "structural MRI" OR "structural MR imaging" OR sMRI OR "T1-weighted MRI" OR "T1 weighted MRI") AND ("deep learning" OR "convolutional neural network" OR "convolutional neural networks" OR CNN OR CNNs OR transformer* OR "self-supervised learning" OR "foundation model" OR "foundation models") |

**Supplementary Table S2. Rationale for the illustrative evidence set**

The 10 works below were chosen purposively to represent distinct technical questions. Recent evidence was prioritized, with one pre-2021 study retained because it is foundational to graph-based population modeling. Selection was qualitative and was not based on citation count, journal metric, or highest reported accuracy.

| **Study and role** | **Why highlighted** | **Claim supported** | **Claim not supported / evidence boundary** |
| --- | --- | --- | --- |
| Parisot et al. (2018) Foundational population GCN | Foundational sMRI example of graph-based population modeling and participant-grouped longitudinal validation. | Supports discussion of relational integration of regional volumes, phenotype-informed edges, and repeated visits. | Single ADNI cohort; regional rather than end-to-end voxel features; graph construction and a semi-supervised/transductive setting constrain generalization and causal interpretation. |
| Goenka and Tiwari (2021) Review / context | Reviewer-requested context for biomarker and input-representation choices. | Frames subject-, ROI-, and patch-level 3D representations and biomarkers. | Heterogeneous modalities and tasks preclude a direct sMRI architecture ranking. |
| Kang et al. (2021) 2D CNN | Illustrates multi-slice transfer learning and ensemble design. | Supports participant classification by aggregating selected 2D slices. | Internal ADNI-1 evidence; selected slices and preprocessing limit whole-volume and external claims. |
| Zhu et al. (2021) 3D attention / multi-instance | Large two-dataset study of participant-level aggregation of local 3D evidence. | Supports four binary tasks, held-out ADNI testing, and independent AIBL evaluation. | Retrospective cohorts; attention is associative; clinical calibration and prospective utility were not established. |
| Lian et al. (2022) Hybrid 3D | Links weak regional localization with multilevel feature fusion. | Supports ADNI-1 development with testing on ADNI-2 and AIBL. | Retrospective testing does not establish calibration or prospective utility; learned regions require biological corroboration. |
| Goenka and Tiwari (2023) Multimodal 3D comparison | Reviewer-requested within-pipeline comparison of subject-, patch-, and reconstructed-slice inputs. | Supports representation trade-offs within one fixed multimodal pipeline. | MRI and PET effects are entangled, and evaluation was internal to ADNI. The 4,315-image/843-participant figures in the article describe Gupta et al. (2013), not the Goenka–Tiwari cohort; no independent participant denominator is asserted here. |
| Chattopadhyay et al. (2024) Architecture / biomarker comparison | Compares CNN, hybrid, and transformer variants for amyloid positivity and reports model-specific samples. | Supports a task-specific comparison and participant-excluded transformer testing, not an architecture-only comparison. | Samples differ by model family; amyloid positivity is not clinical AD diagnosis; evidence is retrospective and ADNI-only. |
| Dhinagar et al. (2025) PEFT / masked autoencoder | Tests resource-efficient adaptation of a brain-MRI pretrained model. | Supports PEFT comparison with full fine-tuning and training from scratch. | Conference-proceedings evidence with 258 AD training scans; no external clinical or calibration evidence. |
| Lin et al. (2025) Vision–language foundation model | Shows an AD-focused foundation model for sMRI plus clinical data. | Supports cross-modal classification, MMSE prediction, selected longitudinal and subgroup analyses, and HABS-HD zero-shot evaluation. | The 3D DenseNet encoder is not a transformer; internal participant-disjoint splitting was unspecified; longitudinal analysis selected correctly classified scans; evidence is retrospective. |
| Bricaud and Masala (2026) Architecture benchmark | Compares 15 architectures within one preprocessing pipeline. | Supports only the pipeline-specific advantage of selected CNN/hybrid models over stand-alone transformers. | Scan-based splitting could place visits from one person across partitions; external and prospective generalization were not established. |
